# Attributes of residual neural networks for modeling fractional differential equations

**DOI:** 10.1016/j.heliyon.2024.e38332

**Published:** 2024-09-26

**Authors:** Sneha Agarwal, Lakshmi Narayan Mishra

**Affiliations:** Department of Mathematics, School of Advanced Sciences, Vellore Institute of Technology, Vellore 632 014, Tamil Nadu, India

**Keywords:** 68T07, 26A33, 47J30, Fractional integral and differential equations, ResNets, Variational iteration approach, Approximate solutions

## Abstract

This paper offers a pioneering in-depth exploration of applying residual neural networks to approximate Erdélyi-Kober fractional derivatives and establishes a parameter upper bound for these networks. We validate this method using the variational iteration formula to obtain the exact solution of a differential equation. The resulting structure from the variational iteration method serves as a basis for showcasing how residual neural networks can effectively estimate these equations. Furthermore, we provide illustrative examples to elucidate the application of residual neural networks in solving equations involving Erdélyi-Kober fractional derivatives.

## 1 Introduction

Due to the widespread use of fractional derivative operators in the mathematical modeling of numerous real-world phenomena that involve nonlocality and memory properties, the theory of fractional calculus has recently received a lot of attention. They can be used to represent sophisticated physical and mechanical processes [32], as well as memory properties in the temporal and spatial domains, path dependence, global importance, and create constitutive models for the behavior of complex mechanics. Recent years have seen a major increase in interest in the theory of fractional integral equations, which is now a significant field of nonlinear analysis [29], [30]. Numerous fractional derivative operators have been suggested and used in the literature, including the Riemann-Liouville, Hadamard [12], Caputo, and Erdélyi-Kober fractional operators [9]. It is challenging to solve fractional differential equations. Fractional calculus has emerged as a powerful framework, offering a suite of innovative techniques for tackling and resolving differential and integral equations [6], [8], thereby unlocking new avenues for problem-solving in various mathematical and scientific domains. For the most part, the Adomian decomposition method [10], collocation method, differential transform method [23], VI approach [35], and others have been developed for the solution of differential equations of fractional order. The Lagrange multiplier method and Newton's method [4] are both considered generalizations of the VI approach. It behaves well for integer differential and integral equations such as the Volterra integral equation [7], the Burger equation [37], the Riccati differential equation [19], the delay differential equation [21], and other relevant equations. It also works efficiently with FDEs, including those with the Riesz sense, the left Riemann-Liouville derivative, the right and left Caputo fractional derivatives [16], fractional third-order dispersive partial differential equations [11], and the Korteweg-de Vries equations with the local fractional derivatives [18].

We can now design a neural network, even one with a high structural complexity, with just a few lines of code due to open source machine learning software libraries like TensorFlow [31], Keras, or PyTorch. Having said that, some of us are still mystified by the mathematics underlying neural networks, and understanding the mathematics underlying deep learning can help us comprehend what goes on within a neural network. Additionally, it helps with architecture selection [36], deep learning model fine-tuning, adjustment of the hyperparameters, and optimization. There has been a growing interest among researchers in applying neural network techniques [25] to solve fractional differential equations, leading to a surge in related publications and studies in recent times [2]. The neural network is used to approximate the unknown function in the equation. A deep FDEs neural network [17] architecture has been proposed for solving FDEs accurately. It has been proven that deep artificial neural networks Jentzen et al. [20] can approximate generic models such as nonlinear drift coefficients and Kolmogorov partial differential equations with constant diffusion and that artificial neural networks Gonon et al. [13] can approximate general heat equations. The architecture uses a Gaussian integration rule and an L1 discretization technique. The ANNs approach boasts the capacity to generate continuous functions that effectively approximate solutions to differential equations, featuring adaptable parameters that enable users to fine-tune the level of accuracy to suit their needs. By adjusting these parameters, the ANNs methodology can produce continuous functions that accurately represent solutions to differential equations with a high degree of precision [3], [22]. The radial basis function method is utilized to solve the initial boundary values of fractional partial differential equations [33] with variable coefficients on a finite domain. ResNets is a deep learning model that was introduced in 2015 by He, Zhang, Ren, and Sun who conducted their research on deep residual learning for image recognition [15]. The ResNets architecture is designed to solve the problem of vanishing gradient in deep neural networks [17], which hindered their performance on large scale image recognition task. In ResNets, the weight layers learn residual functions with reference to the layer inputs. The layer are reformulated as learning residual functions with reference to the layer inputs, instead of learning unreferenced functions. This reformulation makes it easier to optimize the network and can gain accuracy from considerably increased depth.

The following FDE is considered:

(1.1) Dβγ,δ(Dγ,δcϑ)(z)+ϑ(z)=−f(z),z∈[0,T],ϑ(0)=ϑ(T)=0,

where δ∈[1/2,1) and $D_{\beta }^{\gamma ,\delta }$, Dγ,δc denote the EKFD and Caputo type EKFD, respectively. The paper demonstrates that ResNets effectively approximates the solution of Eq.(1.1). ResNets enhances the robustness and generalization capacity of neural networks as well as enabling a faster training method. It provides this by efficiently resolving the network degradation problems and vanishing gradient imposed by growing network depth. To the best of our knowledge, this research work is the first to provide evidence for numerical approximation using deep learning for FDEs. ResNets is a popular technique for solving both linear and nonlinear differential equations, and its capacity to provide imprecise solutions of Eq.(1.1) is demonstrated using the VI method. Its main advantages are convenience and flexibility in solving nonlinear problems [34]. Through VI [27], we will discover the relevant theorems of FDEs with EKFD. Furthermore, our research shows that *X* (the amount of selected points in the specifying region) and *N* (the number of iterations of the estimate solution using the VI approach) are the number of parameters required by ResNets to approximate the solution using polynomial growth factors of Eq.(1.1).

This article is structured as follows: We present three vital theorems, divided between two sections. Section 2 introduces two theorems that explore key properties of fractional calculus and LT, which will be applied in this study. Section 3 then presents a third theorem, focusing on ResNets. Theorem 1 provides the iterative format for solving Eq.(1.1) accurately using the VI method, and shows its convergence to the precise solution. Section 3 demonstrates the existence of a ResNets that can attain the iterated value in Theorem 1, that is, Eq.(1.1) can have a precise solution that the ResNets can approximate. In Section 4, some examples are given to easily understand the results and in the end, we provide the conclusions in Section 5.

We employ the following symbols in the sequel:

•R: Set of all real numbers,

•N: Set of all natural numbers,

•C(R,R): Set of all continuous functions from R→R,

• RV: Restricted variation,

• IM: Identity matrix,

• LT: Laplace transform,

• ResNets: Residual neural networks,

• FDEs: Fractional differential equations,

• EKFD: Erdélyi-Kober fractional differential equations,

• EKFI: Erdélyi-Kober fractional integral equations,

• VI: Variational iteration,

• FNNs: Feedforward neural networks,

• ROR: Realization of ResNets.

## 2 Preliminaries and main results

Definition 2.1 ([26]). The left and right EKFI of order *δ* are defined as

$$(I_{\beta }^{\gamma ,\delta }f)(\vartheta )=\vartheta^{-\beta (\gamma +\delta )}\frac{\beta }{\Gamma (\delta )}\int_{0}^{\vartheta }{\left(\vartheta^{\beta }-z^{\beta }\right)}^{\delta -1}z^{\beta (\gamma +1)-1}f(z)dz,\;\delta ,\beta >0,\gamma \in R,(K_{\beta }^{T,\delta }f)(\vartheta )=\vartheta^{\beta T}\frac{\beta }{\Gamma (\delta )}\int_{\vartheta }^{\infty }{\left(z^{\beta }-\vartheta^{\beta }\right)}^{\delta -1}z^{-\beta (T+\delta -1)-1}f(z)dz,\;\delta ,\beta >0,T\in R,$$

respectively, where $I_{\beta }^{\gamma ,\delta }and\;K_{\beta }^{T,\delta }$ are left and right side EKFI operators.

Definition 2.2 ([26]). The left and right EKFD of order *δ* are defined as

$$(D_{\beta }^{\gamma ,\delta }f)(\vartheta )=\prod_{k=0}^{k=n-1}\left(1+\gamma +k+\frac{\vartheta }{\beta }\frac{d}{d\vartheta }\right)(I_{\beta }^{\gamma +\delta ,n-\delta }f)(\vartheta ),\;\beta >0,\gamma \in R,(P_{\beta }^{T,\delta }f)(\vartheta )=\prod_{k=0}^{k=n-1}\left(T+k-\frac{\vartheta }{\beta }\frac{d}{d\vartheta }\right)(K_{\beta }^{T+\delta ,n-\delta }f)(\vartheta ),\;\beta >0,T\in R,$$

respectively, where (n−1<δ≤n,n∈N) and $I_{\beta }^{\gamma +\delta ,n-\delta }and\;K_{\beta }^{T+\delta ,n-\delta }$ are left and right side EKFI operators, respectively.

Definition 2.3 ([26]). Left and Right Caputo type EKFD (n−1<δ≤n, n∈N)

(Dβγ,δcf)(ϑ)=(Iβγ+δ,n−δ∏k=0k=n−1(1+γ+k+zβddz)f)(ϑ),β>0,γ∈R,(PβT,δcf)(ϑ)=(kβT+δ,n−δ∏k=0k=n−1(T+k−zβddz)f)(ϑ).β>0,γ∈R,

where, Dβγ,δc and PβT,δc are left and right Caputo type EKFD operators, respectively.

Remark 2.4 ([14]). If n=1, then (Dβγ,δcf)(ϑ) can be expressed as

(Dβγ,δcf)(ϑ)=(Iβγ+δ,1−δ(1+γ+zβddz)f)(ϑ)=βΓ(1−δ)ϑ−β(γ+1)∫0ϑ(ϑβ−zβ)−δzβ(γ+δ+1)−1((1+γ+zβddz)f)(z)dz=β(1+γ)Γ(1−δ)ϑ−β(γ+1)∫0ϑ(ϑβ−zβ)−δzβ(γ+δ+1)−1f(z)dz+1Γ(1−δ)ϑ−β(γ+1)∫0ϑ(ϑβ−zβ)−δzβ(γ+δ+1)ddzf(z)dz.

Therefore,

(Dβγ,δcf)(ϑ)−1Γ(1−δ)ϑ−β(γ+1)∫0ϑ(ϑβ−zβ)−δzβ(γ+δ+1)ddzf(z)dz=β(1+γ)Γ(1−δ)ϑ−β(γ+1)∫0ϑ(ϑβ−zβ)−δzβ(γ+δ+1)−1f(z)dz.

Now we use EKFD by putting k=0 and n=1,

(Dβγ,δf)(ϑ)=(1+γ+ϑβddϑ)(Iβγ+δ,1−δf)(ϑ)=(1+γ+ϑβddϑ)(βΓ(1−δ)ϑ−β(γ+1)∫0ϑ(ϑβ−zβ)−δzβ(γ+δ+1)−1f)(z)dz)=(Dβγ,δcf)(ϑ)+ϑΓ(1−δ)[(∫0ϑ(ϑβ−zβ)−δzβ(γ+δ+1)−1f(z)dz)(−β(1+γ))ϑ−β(1+γ)−1+ϑ−β(γ+1)ddϑ∫0ϑ(ϑβ−zβ)−δzβ(γ+δ+1)−1f)(z)dz)]=(Dβγ,δcf)(ϑ)+ϑΓ(1−δ)[(∫0ϑ(ϑβ−zβ)−δzβ(γ+δ+1)−1f)(z)dz)(−β(1+γ))ϑ−β(1+γ)−1]+ϑΓ(1−δ)ϑ−β(1+γ)∫0ϑ−δ(ϑβ−zβ)−δ−1βϑβ−1zβ(γ+δ+1)−1f)(z)dz)

(2.1) (Dβγ,δf)(ϑ)=(Dβγ,δcf)(ϑ)−β1+γΓ(1−δ)ϑ−β(1+γ)∫0ϑ(ϑβ−zβ)−δzβ(γ+δ+1)−1f)(z)dz−βδϑ−βγΓ(1−δ)∫0ϑ(ϑβ−zβ)−δ−1zβ(γ+δ+1)−1f)(z)dz.

Hence, using Eq.(2.1), we get

(2.2) (Dβγ,δf)(ϑ)=(Dβγ,δcf)(ϑ)+W(z)+X(z),

where,

$$W(z)=-\beta \frac{1+\gamma }{\Gamma (1-\delta )}\vartheta^{-\beta (1+\gamma )}\int_{0}^{\vartheta }{\left(\vartheta^{\beta }-z^{\beta }\right)}^{-\delta }z^{\beta (\gamma +\delta +1)-1}f)(z)dz,X(z)=-\frac{\beta \delta \vartheta^{-\beta \gamma }}{\Gamma (1-\delta )}\int_{0}^{\vartheta }{\left(\vartheta^{\beta }-z^{\beta }\right)}^{-\delta -1}z^{\beta (\gamma +\delta +1)-1}f)(z)dz.$$

Definition 2.5 ([28]). LT of the term (Dβγ,δcU)(z) is given as

La[(Dβγ,δcU)(z)]=tδU¯(t)−∑i=0i=n−1Ui(0+)tδ−1−i,n−1<δ≤n,

and, LT is denoted by $L_{a}$, $\overset{¯}{U}(t)=L_{a}[U(z)]$.

Consider $L_{a}[p(z)]=\overset{¯}{p}(t)$ and $L_{a}[q(z)]=\overset{¯}{q}(t)$, the convolution theorem is

$$p(z)⁎q(z)=\int_{0}^{z}p(z-\varsigma )q(\varsigma )d\varsigma ,$$

and

$$L_{a}[p(z)⁎q(z)]=L_{a}[p(z)]L_{a}[q(z)]=\overset{¯}{p}(t)\overset{¯}{q}(t).$$

We generate a new equation by converting Eq.(1.1) to:

(2.3) {(Dβγ,δcϑ)(z)−q(z)=0,(a)(Dβγ,δq)(z)+ϑ(z)+f(z)=0,(b)ϑ(0)=ϑ(T)=0.

The important theorems of this paper are now introduced.

Theorem 1 The exact solutions of the VI formula of ϑ(z) and q(z) for Eq.(2.3)(a) and (2.3)(b), respectively, are

(2.4) ϑn+1(z)=ϑn(z)−0Izδ[Dβδcϑn(ς)−qn(ς)],

(2.5) $$q_{n+1}(z)=q_{n}(z){-}_{z}I_{T}^{\delta }[D_{\beta }^{\delta }q_{n}(\varsigma )+\vartheta_{n}(\varsigma )+f(\varsigma )].$$

Proof The VI process can be applied in both confined and unbounded domains to obtain the exact solution or convergent successive approximation of the differential equations. Firstly, we demonstrate that Eq.(2.4) is the VI formula for ϑ(z). VI method ([35]) is used to attain the approximated solutions of FDEs containing Caputo derivative.

Define U(z) on the set [0,T]. $L_{r}$ and $N_{r}$ stand for linear and nonlinear operators, respectively. If the correctional functional for

(2.6) DβδcU(z)+Lr[U(z)]+Nr[U(z)]=f(z)

is established via left EKFI

Un+1(z)=Un(z)+0Izδλ(z,ς)[DβδcUn(ς)+Lr[Un(ς)]+Nr[Un(ς)]−f(ς)],

where the term $L_{r}[U_{n}]$ and $N_{r}[U_{n}]$ are RV. One way to identify the Lagrange multiplier is λ(z,ς)=−1, and Eq.(2.6) represents iterated formula as

Un+1(z)=Un(z)−0Izδ[DβδcUn(ς)+Lr[Un(ς)]+Nr[Un(ς)]−f(ς)].

One can find the approximate solution $U_{n}(z)$ by using any zeroth approximation $U_{0}(z)=1$. Accordingly, the VI formula for ϑ(z) is

(2.7) ϑn+1(z)=ϑn(z)−0Izδ[Dβδcϑn(ς)−qn(ς)].

Now, we show that VI formula of q(z) is Eq.(2.5). In the correction functional of the equation below, we need to get the Lagrange multiplier.

Consider the following equation:

(2.8) $$D_{\beta }^{\delta }U(z)+L_{r}[U(z)]+N_{r}[U(z)]=f(z),$$

where z∈[0,T]. Writing EKFD in terms of Caputo EKFD from Eq.(2.2), we have

$$D_{\beta }^{\delta }U(z)={}_{c}D_{\beta }^{\delta }U(z)+W(z)+X(z),$$

where,

$$W(z)=-\beta \frac{1+\gamma }{\Gamma (1-\delta )}\vartheta^{-\beta (1+\gamma )}\int_{0}^{\vartheta }{\left(\vartheta^{\beta }-z^{\beta }\right)}^{-\delta }z^{\beta (\gamma +\delta +1)-1}f)(z)dz,X(z)=-\beta \frac{\delta \vartheta^{-\beta \gamma }}{\Gamma (1-\delta )}\int_{0}^{\vartheta }{\left(\vartheta^{\beta }-z^{\beta }\right)}^{-\delta -1}z^{\beta (\gamma +\delta +1)-1}f)(z)dz.$$

Now, EKFI is used to determine the correctional functional for 0<δ<1 in Eq.(2.8).

(2.9) Un+1(z)=Un(z)+zITδλ(z,ς)[DβδcUn(z)+Wn(ς)+Xn(ς)+Lr[Un(ς)]+Nr[Un(ς)]−f(ς)],

subsequently, the Lagrange multiplier is known as λ(z,ς)=−1, where the terms $W_{n},X_{n},L_{r}[U_{n}]$ and $N_{r}[U_{n}]$ are RV. Applying LT in Eq.(2.9), we obtain

(2.10) U¯n+1(t)=U¯n(t)+La[ITδzλ(z,ς)[DβδcUn(ς)+Wn(ς)+Xn(ς)+Lr[Un(ς)]+Nr[Un(ς)]−f(ς)]].

Now, we have

ITδzλ(z,ς)[DβδcUn(ς)]=βΓ(δ)ϑ−β(γ+δ)∫zT(ϑβ−zβ)δ−1λ(z,ς)zβ(γ+1)−1cDβδUn(ς)dς=βΓ(δ)ϑ−β(γ+δ)∫0T(ϑβ−zβ)δ−1λ(z,ς)zβ(γ+1)−1cDβδUn(ς)dς−βΓ(δ)ϑ−β(γ+δ)∫0z(ϑβ−zβ)δ−1λ(z,ς)zβ(γ+1)−1cDβδUn(ς)dς=F(z)−βΓ(δ)ϑ−β(γ+δ)∫0z(ϑβ−zβ)δ−1λ(z,ς)zβ(γ+1)−1cDβδUn(ς)dς=F(z)−βΓ(δ)ϑ−β(γ+δ)∫0z(−1)δ−1(zβ−ϑβ)δ−1λ(z−ς)zβ(γ+1)−1cDβδUn(ς)dς=F(z)−(−1)δ−1λ(z)Γ(δ)(zβ)δ−1zβ(γ+1)−1⁎cDβδUn(z)=F(z)−(−1)δ−1λ(z)Γ(δ)zβ(δ+γ)−1⁎cDβδUn(z)=F(z)−μ(z)⁎cDβδUn(z).F(z)=βΓ(δ)ϑ−β(γ+δ)∫0T(ϑβ−zβ)δ−1λ(z,ς)zβ(γ+1)−1cDβδUn(ς)dςμ(z)=(−1)δ−1λ(z)Γ(δ)zβ(δ+γ)−1.λ(z,ς)=λ(z−ς)La[ITδzλ(z,ς)cDβδUn(z)]=La[F(z)−μ(z)⁎cDβδUn(z)]=La[F(z)]−La[μ(z)]⋅La[DzδcUn(z)]=La[F(z)]−μ¯(t)⋅[tδU¯n(t)−Un(0+)tδ−1].

Applying the derivative of the classical variation Δ to each side of Eq.(2.10). As $W_{n}$, $X_{n}$, $L_{r}[U_{n}]$ and $N_{r}[U_{n}]$ are referred as RV, F(z) is not the functioning component of $U_{n}(z)$, Eq.(2.10) can be computed as

$$\Delta {\overset{¯}{U}}_{n+1}(t)=\Delta {\overset{¯}{U}}_{n}(t)+\Delta [-\overset{¯}{\mu }(t)[t^{\delta }{\overset{¯}{U}}_{n}(t)-U_{n}(0^{+})t^{\delta -1}]]=[1-\overset{¯}{\mu }(t)t^{\delta }]\Delta {\overset{¯}{U}}_{n}(t)1-\overset{¯}{\mu }(t)t^{\delta }=0\;\overset{¯}{\mu }(t)=\frac{1}{t^{\delta }}\;\mu (z)={\left(-1\right)}^{\delta }\frac{z^{\delta -1}}{\Gamma (\delta )}.$$

Thus, Eq.(2.8) shows the iteration formula as

Un+1=Un−zITδ[DβδcUn(ς)+Wn(ς)+Xn(ς)+Lr[Un(ς)]+Nr[Un(ς)]−F(ς)]=Un−zITδ[DβδUn(ς)+Lr[Un(ς)]+Nr[Un(ς)]−F(ς)].

Accordingly the VI formula for q(z) is

(2.11) $$q_{n+1}(z)=q_{n}(z){-}_{z}I_{T}^{\delta }[D^{\delta }q_{n}(\varsigma )+\vartheta (\varsigma )+F(\varsigma )].$$

Obtaining Eq.(2.7) and (2.11), that is, acquiring the VI formulas of ϑ(z) and q(z), respectively, shows the proof of Theorem 1. □

Theorem 2 Let $\vartheta_{0}(z)$ = Φ(z) and Φ(0) = ϑ(0). The sequence of $\left\{\vartheta_{n}(z)\right\}$ and $\left\{q_{n}(z)\right\}$ obtained using Theorem 1 converges to the precise values ϑ(z) and q(z).

Proof The section states that how the sequences of $\left\{\vartheta_{n}(z)\right\}$ and $\left\{q_{n}(z)\right\}$, which were derived in Theorem 1, converges to the precise solution of ϑ(z) and q(z).

It can be obtained as:

ϑ(z)=ϑ(z)−0Izδ[Dβδcϑ(ς)−q(ς)],q(z)=q(z)−zITδ[Dβδq(ς)+ϑ(ς)+F(ς)].

Let

$$e_{n}(z)=\vartheta_{n}(z)-\vartheta (z),\epsilon_{n}(z)=q_{n}(z)-q(z),$$

we can obtain that,

(2.12) en+1(z)=en(z)−0Izδ[Dβδcen(ς)−ϵn(ς)],

(2.13) $$\epsilon_{n+1}(z)=\epsilon_{n}(z){-}_{z}I_{T}^{\delta }[D_{\beta }^{\delta }\epsilon_{n}(\varsigma )+e_{n}(\varsigma )],$$

where

δ∈(0,1),Izδ0[Dβδcϑ(z)]=ϑ(z)−ϑ(0),ITδz[Dβδϑ(z)]=ϑ(z)−zIT1−δϑ(z)|z=T,

then

$${|}_{z}I_{T}^{\delta ,1-\gamma }f(t)|=|\frac{\beta }{\Gamma (1-\delta )}t^{-\beta (\gamma +1-\delta )}\int_{z}^{T}{\left(t^{\beta }-z^{\beta }\right)}^{-\delta }z^{\beta (\gamma +1)-1}f(z)dz|,\leq \max|f(z)|\frac{\beta }{\Gamma (1-\delta )}t^{-\beta (\gamma +1-\delta )}\int_{z}^{T}{\left(t^{\beta }-z^{\beta }\right)}^{-\delta }z^{\beta (\gamma +1)-1}dz.$$

Let γ=0, thus

$${|}_{z}I_{T}^{\delta ,1-\gamma }f(t)|\leq \max|f(z)|\frac{\beta }{\Gamma (1-\delta )}t^{-\beta (1-\delta )}\int_{z}^{T}{\left(t^{\beta }-z^{\beta }\right)}^{-\delta }z^{\beta -1}dz.$$

Let $t^{\beta }-z^{\beta }=u;-\beta z^{\beta -1}dz=du$, therefore,

$${|}_{z}I_{T}^{\delta ,1-\gamma }f(t)|\leq \max|f(z)|\frac{\beta }{\Gamma (1-\delta )}(-t^{-\beta (1-\delta )})\int \frac{u^{-\delta }}{-\beta }du\leq \max\frac{\left|f(z)\right|}{\Gamma (1-\delta )}(-t^{-\beta (1-\delta )}){\left[\frac{{\left(t^{\beta }-z^{\beta }\right)}^{1-\delta }}{1-\delta }\right]}_{z}^{T}\leq \max\frac{\left|f(z)\right|}{\Gamma (1-\delta )}\frac{t^{-\beta (1-\delta )}}{\left(1-\delta \right)}[{\left(t^{\beta }-T^{\beta }\right)}^{1-\delta }-{\left(t^{\beta }-z^{\beta }\right)}^{1-\delta }],$$

when $z=T,\;{|}_{z}I_{T}^{1-\delta }\vartheta (z)|\leq 0$, which represents IT1−δzϑ(z)|(z=T)=0.

It is simple to prove that $e_{n}(0)=0$, $\epsilon_{n}(z)$ is bounded on [0,T]. Then Eq.(2.12) and (2.13) can be reduced to

$$e_{n+1}(z){=}_{0}I_{z}^{\delta }[\epsilon_{n}(\varsigma )],\epsilon_{n+1}(z)={-}_{z}I_{T}^{\delta }[e_{n}(\varsigma )].$$

Solving $e_{n+1}(z)$ and $\epsilon_{n+1}(z)$ for their absolute values, we obtain

$$|e_{n+1}(z)|={|}_{0}I_{z}^{\delta }[e_{n}(\varsigma )]|=|\frac{\beta }{\Gamma (\delta )}z^{-\beta (\gamma +\delta )}\int_{0}^{z}{\left(z^{\beta }-\varsigma^{\beta }\right)}^{\delta -1}\varsigma^{\beta (\gamma +1)-1}|\epsilon_{n}(\varsigma )|d\varsigma |\leq \frac{\beta }{\Gamma (\delta )}z^{-\beta (\gamma +\delta )}\int_{0}^{z}{\left(z^{\beta }-\varsigma^{\beta }\right)}^{\delta -1}\varsigma^{\beta (\gamma +1)-1}\max|\epsilon_{n}(\varsigma )|d\varsigma \leq_{0}I_{z}^{\delta }\|\epsilon_{n}\|,\|e_{n+1}\|=\max_{z\in [0,T]}|e_{n+1}|\leq {\max_{z\in [0,T]}}_{0}I_{z}^{\delta }\|\epsilon_{n}\|=\max_{z\in [0,T]}\|\epsilon_{n}\|\int_{0}^{z}{\left(z^{\beta }-\varsigma^{\beta }\right)}^{\delta -1}\varsigma^{\beta -1}d\varsigma .$$

Let $z^{\beta }-\varsigma^{\beta }=u$; $-\beta \varsigma^{\beta -1}d\varsigma =du$, thus

$$\|e_{n+1}\|=\max\|\epsilon_{n}\|\int \frac{u^{\delta -1}}{-\beta }du=\max\frac{\left\|\epsilon_{n}\right\|}{-\beta \delta }{\left[{\left(z^{\beta }-\varsigma^{\beta }\right)}^{\delta }\right]}_{0}^{z}=\max\frac{\left\|\epsilon_{n}\right\|}{\beta \delta }[{\left(z^{\beta }\right)}^{\delta }]{=}_{0}I_{T}^{\delta }\|\epsilon_{n}\|,$$

and

|ϵn+1(z)|=|−zITδ(en(ς))|≤zITδ‖en(ς)‖‖ϵn+1‖=maxt∈[0,T]⁡|ϵn+1(z)|≤maxz∈[0,T]z⁡ITδ‖en‖=maxz∈[0,T]⁡‖en‖Γ(δ)z−β(γ+δ)(Tβ−ςβ)δβδ=‖en‖Γ(δ)(Tβ)δβδ=0ITδ‖en‖=aIzδ1[Izδ2af(z)]=aIzδ1+δ2f(z),

holds for any δ,β≥0,γ=0,∀ϵ∈[a,b], if function *f* is continuous,

(2.14) $$\|e_{n+1}\|+\|\epsilon_{n+1}\|\leq_{0}I_{T}^{\delta_{1}}\|\epsilon_{n}\|+{}_{0}I_{T}^{\delta_{1}}\|e_{n}\|{=}_{0}I_{T}^{\delta_{1}}[\|e_{n}\|+\|\epsilon_{n}\|]\leq_{0}I_{T}^{2\delta_{1}}[\|e_{n-1}\|+\|\epsilon_{n-1}\|]\leq \cdots \leq_{0}I_{T}^{(n+1)\delta_{1}}[\|e_{0}\|+\|\epsilon_{0}\|]=\frac{\left\|e_{0}\|+\|\epsilon_{0}\right\|}{\Gamma (n\delta_{1}+\delta_{1})}\beta T^{-\beta (\gamma +\delta_{1})}\int_{0}^{T}{\left(T^{\beta }-t^{\beta }\right)}^{n\delta_{1}+\delta_{1}-1}t^{\beta (\gamma +1)-1}dt=\frac{\left\|e_{0}\|+\|\epsilon_{0}\right\|}{\Gamma (n\delta_{1}+\delta_{1})}\beta T^{-\beta \delta_{1}}\int_{0}^{T}{\left(T^{\beta }-t^{\beta }\right)}^{n\delta_{1}+\delta_{1}-1}t^{\beta -1}dt.=\frac{\left\|e_{0}\|+\|\epsilon_{0}\right\|}{\Gamma (n\delta_{1}+\delta_{1})}\frac{T^{-\beta \delta_{1}}}{n\delta_{1}+\delta_{1}}{\left(T^{\beta }\right)}^{n\delta_{1}+\delta_{1}}.$$

On comparing above expression with the formula [1], $\Gamma (n\alpha_{1}+\alpha_{1})∼\sqrt{2\pi }\exp(-n\alpha_{1}){\left(n\alpha_{1}\right)}^{n\alpha_{1}+\alpha_{1}-1/2}$, then

(2.15) $$\frac{1}{\Gamma (n\delta_{1}+\delta_{1})}\frac{{\left(T^{\beta }\right)}^{n\delta_{1}}}{\left(n\delta_{1}+\delta_{1}\right)}∼\frac{T^{n\delta_{1}\beta }}{\sqrt{2\pi }\exp(-n\delta_{1}){\left(n\delta_{1}\right)}^{n\delta_{1}+\delta_{1}-1/2}}\frac{1}{n\delta_{1}+\delta_{1}},$$

combining Eq.(2.14) and (2.15), we get

$$\max_{z\in [0,T]}(|e_{n+1}(z)|+|\epsilon_{n+1}(z)|)\leq \|e_{n+1}\|+\|\epsilon_{n+1}\|\leq \frac{\left\|e_{0}\|+\|\epsilon_{0}\right\|}{\sqrt{2\pi }{\left(n\delta_{1}\right)}^{n\delta_{1}+\delta_{1}}}\frac{\exp(n\delta_{1})T^{n\delta_{1}\beta }{\left(n\delta_{1}\right)}^{1/2}}{\left(n\delta_{1}+\delta_{1}\right)},$$

since $\left\|e_{0}\right\|$ + $\left\|\epsilon_{0}\right\|$ and *T* are constants, as n→∞, we have

$$\lim_{n\to \infty }\max_{z\in [0,T]}(|e_{n+1}(z)|+|\epsilon_{n+1}(z)|)\leq \lim_{n\to \infty }\frac{\left\|e_{0}\|+\|\epsilon_{0}\right\|}{\sqrt{2\pi }{\left(n\delta_{1}\right)}^{n\delta_{1}+\delta_{1}}}\frac{\exp(n\alpha_{1})T^{n\delta_{1}\beta }{\left(n\delta_{1}\right)}^{1/2}}{\left(n\delta_{1}+\delta_{1}\right)}\leq \frac{\left\|e_{0}\|+\|\epsilon_{0}\right\|}{\sqrt{2\pi }}\lim_{n\to \infty }\frac{\exp(n\delta_{1})T^{n\delta_{1}\beta }{\left(n\delta_{1}\right)}^{1/2}}{(n\delta_{1}+\delta_{1}){\left(n\delta_{1}\right)}^{n\delta_{1}+\delta_{1}}}=0.$$

This shows that the sum of the absolute values of the errors between $\vartheta_{n}$(z) and ϑ(z), and the errors between $q_{n}(z)$ and q(z) converges to 0 when *n*→ ∞. Consequentially, the proof of Theorem 2 is complete. □

## 3 ResNets based approximation

After Theorem 2 has been verified, we represent $\vartheta_{N}(z)$ and $q_{N}(z)$ as the approximate solutions of Eq. (2.3)(a) and Eq.(2.3)(b), respectively. Next, we discuss a few definitions and lemmas in Section 3.1 related to FNNs and ResNets.

### 3.1 FNNs and ResNets

We begin by defining FNNs and outlining some characteristics.

Definition 3.1 (FNNs [20]). The collection of all FNNs is referred to as

$$F=\underset{S\in N}{⋃}\underset{\ell_{0},\ell_{1}\cdots \ell_{S}\in N}{⋃}(\times_{k=1}^{S}(R^{\ell_{k}\times \ell_{k-1}}\times R^{\ell_{k}})),$$

and $P_{c},L_{1},O,I_{d}:F\to N,C:F\to ({⋃}_{S\in N}N^{S}),D_{n}:F\to N_{0}$ are known as the functions which satisfy for $S\in N,\ell_{0},\ell_{1},\cdots ,\ell_{S}\in N,⊖=((A_{1},B_{1}),(A_{2},B_{2}),\cdots ,(A_{S},B_{S}))\in (\times_{k=1}^{S}(R^{\ell_{k}\times \ell_{k-1}}\times R^{\ell_{k}}))$ such that $I_{d}(⊖)=\ell_{0}$, $L_{1}(⊖)$: = S, $O(⊖)=\ell_{S}$, $P_{c}(⊖)=\sum_{k=1}^{S}\ell_{k}(\ell_{k-1}+1)$, $C(⊖)=(\ell_{0},\ell_{1},\cdots ,\ell_{S})$ and $C_{p}(⊖)=\ell_{p},p=0,1,\cdots ,S$. ⊖ is called an FNNs and $L_{1}(⊖)$ is the depth of ⊖, $\ell_{i}$ is the size of layer i, the input dimension and the output dimension are $I_{d}(⊖)$ and O(⊖) respectively, the complexity of ⊖ is $P_{c}(⊖)$ and $C(⊖),D_{p}(⊖)$ are the architecture of ⊖.

Definition 3.2 (Realizations of FNNs [20]). Let *μ*∈ C(R, R). $F_{\mu }$ is the function which satisfies ∀ $\vartheta_{0}\in R^{\ell_{0}},\vartheta_{1}\in R^{\ell_{1}},\cdots ,\vartheta_{S-1}\in R^{\ell_{S-1}}$

$$(F_{\mu }⊖)(\vartheta_{0})=A_{S}\vartheta_{S-1}+B_{S},$$

where $\vartheta_{k}=M_{\mu }(A_{k}\vartheta_{k-1}+B_{k}),k=1,\cdots ,S-1.\;F_{\mu }⊖\in C(R^{\ell_{0}},R^{\ell_{S}}).\;F_{\mu }⊖$ is the function of ⊖, *μ* is referred to as the activation function.

Lemma 3.3 *(**[20]**). If*

(⋅) • (⋅)*: {*$\left(\theta_{1},\theta_{2})\in F\times F|I_{d}(\theta_{2}\right)$*=*$O(\theta_{1})\}\to F$ *referred to as the function that satisfies for*

$$\theta_{1}=((W_{1},B_{1}),(W_{2},B_{2}),\cdots ,(W_{L_{1}},B_{L_{1}}))$$

$$\theta_{2}=((A_{1},B_{1}),(A_{2},B_{2}),\cdots ,(A_{S},B_{S}))$$

*such that*

$$\theta_{2}•\theta_{1}=((W_{1},B_{1}),(W_{2},B_{2}),\cdots ,(W_{L_{1}-1},B_{L_{1}-1}),(A_{1}W_{L_{1}},A_{1}B_{L_{1}}+B_{1}),(A_{2},B_{2}),\cdots ,(A_{S},B_{S})),$$

*then*

$$C(\theta_{2}•\theta_{1})=(D_{0}(\theta_{1}),D_{1}(\theta_{1}),\cdots ,D_{L_{1}(\theta_{1})-1}(\theta_{1}),D_{1}(\theta_{2}),D_{2}(\theta_{2}),\cdots ,D_{L_{1}(\theta_{2})}(\theta_{2})),$$

$$F_{\mu }(\theta_{2}•\theta_{1})=[F_{\mu }(\theta_{2})]•[F_{\mu }(\theta_{1})].$$

Lemma 3.4 *(**[20]**). Let*p∈N*, then*

$$P_{p}:(\theta_{1},\theta_{2},\cdots ,\theta_{p})\in F^{p}|L_{1}(\theta_{1})=L_{1}(\theta_{2})=\cdots =L_{1}(\theta_{p})\to F$$

*is defined by the functions*

$$\theta_{1}=((A_{1}^{1},B_{1}^{1}),(A_{2}^{1},B_{2}^{1}),\cdots ,(A_{S}^{1},B_{S}^{1})),\theta_{2}=((A_{1}^{2},B_{1}^{2}),(A_{2}^{2},B_{2}^{2}),\cdots ,(A_{S}^{2},B_{S}^{2})),\vdots \;\theta_{n}=((A_{1}^{p},B_{1}^{p}),(A_{2}^{p},B_{2}^{p}),\cdots ,(A_{S}^{p},B_{S}^{p})).$$

$$P_{p}(\theta_{1},\theta_{2},\cdots ,\theta_{p})=(\left(\left[\begin{array}{cccc} A_{1}^{1} & 0 & \cdots & 0\\ 0 & A_{1}^{2} & \cdots & 0\\ \vdots & \vdots & \ddots & \vdots \\ 0 & 0 & \cdots & A_{1}^{p} \end{array}\right],\left[\begin{array}{c} B_{1}^{1}\\ B_{1}^{2}\\ \vdots \\ B_{1}^{p} \end{array}\right]\right),\left(\left[\begin{array}{cccc} A_{2}^{1} & 0 & \cdots & 0\\ 0 & A_{2}^{2} & \cdots & 0\\ \vdots & \vdots & \ddots & \vdots \\ 0 & 0 & \cdots & A_{2}^{p} \end{array}\right],\left[\begin{array}{c} B_{2}^{1}\\ B_{2}^{2}\\ \vdots \\ B_{2}^{p} \end{array}\right]\right),\cdots ,\left(\left[\begin{array}{cccc} A_{S}^{1} & 0 & \cdots & 0\\ 0 & A_{S}^{2} & \cdots & 0\\ \vdots & \vdots & \ddots & \vdots \\ 0 & 0 & \cdots & A_{S}^{p} \end{array}\right],\left[\begin{array}{c} B_{S}^{1}\\ B_{S}^{2}\\ \vdots \\ B_{S}^{p} \end{array}\right]\right)).$$

*Let θ=*$(\theta_{1},\theta_{2},\cdots ,\theta_{p})\in F^{p}$*, then*

$$C(P_{p}(\theta ))=(\sum_{j=1}^{p}D_{0}(\theta_{j}),\sum_{j=1}^{p}D_{1}(\theta_{j}),\cdots ,\sum_{j=1}^{p}D_{L_{1}(\theta_{1})}(\theta_{j})),$$

$$(F_{\mu }(P_{p}(\theta )))(\vartheta_{1},\vartheta_{2},\cdots ,\vartheta_{p})=((F_{\mu }\theta_{1})(\vartheta_{1}),(F_{\mu }\theta_{2})(\vartheta_{2}),\cdots ,(F_{\mu }\theta_{p})(\vartheta_{p})).$$

Lemma 3.5 *(**[20]**). Let θ =*$\left((A_{1},B_{1}),(A_{2},B_{2}),\cdots ,(A_{S},B_{S})\right)$*,*$W\in R^{m\times \ell_{S}}$*,*$W\in R^{\ell_{0}\times n}$*, FNNs*θ⊛W*and*W⊛θ*are respectively defined as*

$$W⊛\theta =((A_{1},B_{1}),(A_{2},B_{2}),\cdots ,(A_{S-1},B_{S-1}),(WA_{S},WB_{S})),$$

$$\theta ⊛W=((A_{1}W,B_{1}),(A_{2},B_{2}),\cdots ,(A_{S},B_{S})),$$

*then*

$$F_{\mu }(W⊛\theta )\in C(R^{I_{d}^{\left(\theta \right)}},R^{m}),F_{\mu }(\theta ⊛W)\in C(R^{n},R^{O(\theta )}),$$

$$(F_{\mu }(W⊛\theta ))(\vartheta )=W(F_{\mu }\theta (\vartheta )),$$

$$F_{\mu }(\theta ⊛W))(\vartheta )=F_{\mu }\theta (W_{\vartheta }).$$

Lemma 3.6 *(**[20]**). Let*$X\in N,h_{1},h_{2},\cdots ,h_{X}\in R,\theta_{1},\theta_{2},\cdots ,\theta_{X}\in F$*satisfy that*$C(\theta_{1})=C(\theta_{2})=\cdots =C(\theta_{X})=(\ell_{0},\ell_{1},\cdots ,\ell_{S})$*. Then*∃ψ∈F*such that*$\forall \;\vartheta \in R^{I_{d}(\theta_{1})}$*it holds that*$F_{\mu }\psi \in C(R^{I_{d}(\theta_{1})},R^{O(\theta_{1})})$*,*$P_{c}(\psi )\leq X^{2}P_{c}(\theta_{1})$*and*

$$(F_{\mu }\psi )(\vartheta )=\sum_{m=1}^{X}h_{X}[(F_{\mu }\theta_{m})(\vartheta )],$$

*where*

$$\psi =A_{2}⊛P_{X}(\theta_{1},\theta_{2},\cdots ,\theta_{X})⊛A_{1},$$

*with*

$$A_{1}=\left[\begin{array}{c} I_{\ell_{0}}\\ \vdots \\ I_{\ell_{0}} \end{array}\right],A_{2}=(h_{1}I_{\ell_{S}}\cdots h_{X}I_{\ell_{S}}).$$

The definition of ResNets and a few of its characteristics are now discussed. FNNs and general ResNets are distinguished by the shortcut connections between different layers.

**Figure 1:**
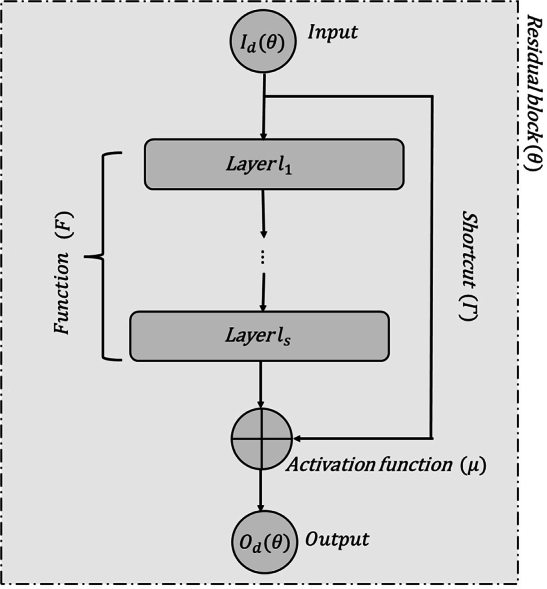
The architecture of the ResNets.

Imagine a series of layers in a neural network shown in Fig. 1, from layer $l_{1}$ to layer $l_{s}$, that collectively perform a function F. Normally, input $I_{d}(\theta )$ would flow through each layer sequentially, resulting in output $O_{d}(\theta )$ at the final layer. However, a residual connection can shortcut (Γ) this process by bypassing these layers, altering the flow of information. The neural network component that takes input $I_{d}(\theta )$ and outputs the sum of F and $I_{d}(\theta )$ is often called a residual block or building block. These residual blocks frequently include an activation function, which is applied to the output $O_{d}(\theta )$ to introduce nonlinear properties.

### 3.2 Definition

(ResNets [5]). The set of all ResNets is defined by

$$S:=\cup_{p\in N}S_{p},$$

where ∀ p∈N,

$$S_{p}:=\cup_{(d_{0},d_{1},\cdots ,d_{p})\in N^{p}}\left\{(\Gamma_{1},\theta_{1},\Gamma_{2},\theta_{2},\cdots ,\Gamma_{p},\theta_{p})\;|\begin{array}{c} \theta_{k}\in F,I_{d}(\theta_{k})=d_{k-1},O(\theta_{k})=d_{k},\\ \Gamma_{k}\in R^{d_{k}\times d_{k-1}},\forall k\in \{1,2,\cdots ,p\} \end{array}\right\}.$$

Let $p\in N,d_{0},d_{1},\cdots ,d_{p}\in N$, and assume that the ResNets *ζ* ∈ S be given by *ζ* = $\left(\Gamma_{1},\theta_{1},\Gamma_{2},\theta_{2},\cdots ,\Gamma_{p},\theta_{p}\right)$,

∀ k ∈{1,2,⋯,p}, we have $I_{d}(\theta_{k})$ = $d_{k-1},O(\theta_{k})=d_{k}$ and $\Gamma_{k}\in R^{d_{k}\times d_{k-1}}$. Let the residual blocks of *ζ* is *θ*= $(\theta_{1},\theta_{2},\cdots ,\theta_{p})\in F^{p}$ and $\Gamma_{k}$ is the shortcut. Furthermore, K,E:S→N, are known as the length and complexity of *ζ* by the functions that fulfill $K(\zeta )=p,E(\zeta )=\sum_{i=1}^{p}(P_{c}(\theta_{i})+d_{i}d_{i-1})$, respectively.

Moreover, we denote $D:S\to \cup_{S\in N}N^{S}$ by the function that satisfies $D(\zeta )=(d_{0},d_{1},\cdots ,d_{K(\zeta )})$.

### 3.3 Definition

(ROR [5]). Consider μ∈C(R,R), and $R_{\mu }$ is identified as the function which holds for every

$$\zeta =(\Gamma_{1},\theta_{1},\Gamma_{2},\theta_{2},\cdots ,\Gamma_{n},\theta_{n})\in S,$$

and $\vartheta_{0}\in R^{I_{d}(\theta_{1})},\vartheta_{1}\in R^{I_{d}(\theta_{2})}=R^{O(\theta_{1})},\cdots ,\vartheta_{n}\in R^{O(\theta_{n})}$ with

$$\vartheta_{i}=\Gamma_{i}\vartheta_{i-1}+F_{\mu }\theta_{i}(\vartheta_{i-1}),$$

for all i∈{1,2,⋯,n} that $(R_{\mu }\zeta )(\vartheta_{0})=\vartheta_{n}$. The activation function is denoted by the symbol *μ* and the function $R_{\mu }\zeta \in C(R^{I_{d}(\theta_{1})},R^{O(\theta_{n})})$ is called the ROR *ζ*.

Lemma 3.7 *(**[5]**). Let*m,n∈N*, the ResNets*$\Theta^{1}=(\Gamma_{1}^{1},\theta_{1}^{1},\Gamma_{2}^{1},\theta_{2}^{1},\cdots ,\Gamma_{n}^{1},\theta_{n}^{1})$*,*

$\Theta^{2}=(\Gamma_{1}^{2},\theta_{1}^{2},\Gamma_{2}^{2},\theta_{2}^{2},\cdots ,\Gamma_{m}^{2},\theta_{m}^{2})$*satisfy*$O(\theta_{n}^{1})=I_{d}(\theta_{1}^{2})$*, the composition of*$\Theta^{1}$*and*$\Theta^{2}$*is defined as*

$$\Theta^{2}•\Theta^{1}=(\Gamma_{1}^{1},\theta_{1}^{1},\Gamma_{2}^{1},\theta_{2}^{1},\cdots ,\Gamma_{n}^{1},\theta_{n}^{1},\Gamma_{1}^{2},\theta_{1}^{2},\Gamma_{2}^{2},\theta_{2}^{2},\cdots ,\Gamma_{m}^{2},\theta_{m}^{2})\in S,$$

*then these two attributes hold*

$$R_{\mu }(\Theta^{2}•\Theta^{1})=R_{\mu }(\Theta^{2})\circ R_{\mu }(\Theta^{1}),$$

$$E_{\mu }(\Theta^{2}•\Theta^{1})=E_{\mu }(\Theta^{1})+E_{\mu }(\Theta^{2}).$$

Lemma 3.8 *(**[5]**). Let*$U,i\in N,\Theta^{j}$*=*$(\Gamma_{1}^{j},\theta_{1}^{j},\Gamma_{2}^{j},\theta_{2}^{j},\cdots ,\Gamma_{i}^{j},\theta_{i}^{j})\in F$*,* ∀ *j* ∈ {1,2,⋯,U}*. Assume that* $C(\theta_{n}^{1})=\cdots =C(\theta_{n}^{U})$*,* ∀ *n* ∈ *{1, 2,* ⋯*, i}. Then* $P_{U}$ *is defined as*

$$P_{U}(\Theta^{1},\Theta^{2},\cdots ,\Theta^{U}):=(\left[\begin{array}{cccc} \Gamma_{1}^{1} & 0 & \cdots & 0\\ 0 & \Gamma_{1}^{2} & \cdots & 0\\ \vdots & \vdots & \ddots & \vdots \\ 0 & 0 & \cdots & \Gamma_{1}^{U} \end{array}\right],P_{U}(\theta_{1}^{1},\theta_{1}^{2},\cdots ,\theta_{1}^{U}),\;\left[\begin{array}{cccc} \Gamma_{2}^{1} & 0 & \cdots & 0\\ 0 & \Gamma_{2}^{2} & \cdots & 0\\ \vdots & \vdots & \ddots & \vdots \\ 0 & 0 & \cdots & \Gamma_{2}^{U} \end{array}\right],P_{U}(\theta_{2}^{1},\theta_{2}^{2},\cdots ,\theta_{2}^{U}),\cdots ,\left[\begin{array}{cccc} \Gamma_{i}^{1} & 0 & \cdots & 0\\ 0 & \Gamma_{i}^{2} & \cdots & 0\\ \vdots & \vdots & \ddots & \vdots \\ 0 & 0 & \cdots & \Gamma_{i}^{U} \end{array}\right],P_{U}(\theta_{i}^{1},\theta_{i}^{2},\cdots ,\theta_{i}^{U})).$$

$P_{U}$ *is still a ResNets. Let* $\vartheta_{0}^{1},\vartheta_{0}^{2},\cdots ,\vartheta_{0}^{U}\in R^{I_{d}(\theta_{1}^{1})}$*, then*

$$R_{\mu }(P_{U}(\Theta^{1},\Theta^{2},\cdots ,\Theta^{U}))(\vartheta_{0}^{1},\vartheta_{0}^{2},\cdots ,\vartheta_{0}^{U})=((R_{\mu }\Theta^{1})(\vartheta_{0}^{1}),(R_{\mu }\Theta^{2})(\vartheta_{0}^{2}),\cdots ,(R_{\mu }\Theta^{U})(\vartheta_{0}^{U})),$$

$$E(P_{U}(\Theta^{1},\Theta^{2},\cdots ,\Theta^{U}))\leq U^{2}E(\Theta^{1}).$$

We will then go over a property in which the collection of the realizations of the individual ResNets that create the original ResNets equals the realization of a parallelized ResNets.

### 3.4 Proposition

([38]). Let U,n∈N and let $\Theta^{j}:=(\Gamma_{1}^{j},\theta_{1}^{j},\Gamma_{2}^{j},\theta_{2}^{j},\cdots ,\Gamma_{n}^{j},\theta_{n}^{j})\in F,j\in \{1,2,\cdots ,U\}$, satisfy $C(\theta_{i}^{1})=C(\theta_{i}^{2})=\cdots ,=C(\theta_{i}^{U}),\forall \;i\in \{1,2,\cdots ,n\}$, that is, the architecture of every $i^{th}$ residual block is the same. Now, a ResNets Ψ∈F, $\forall \;\vartheta \in R^{I_{d}(\theta_{1}^{1})}$,

$$(R_{\mu }\Psi )(\vartheta )=((R_{\mu }\Theta^{1})(\vartheta ),(R_{\mu }\Theta^{2})(\vartheta ),\cdots ,(R_{\mu }\Theta^{U})(\vartheta )),$$

and the property is satisfied by the complexity of Ψ, $E(\Psi )\leq U^{2}E(\Theta^{1})$.

Proof Let $d_{0},d_{1},\cdots ,d_{n}\in N$, ∀ j∈{1,2,⋯,U}, $D(\Theta^{j})$ = $\left(d_{0},d_{1},\cdots ,d_{n}\right)$. Set

$$\Psi :=(\left[\begin{array}{cccc} \Gamma_{1}^{1} & 0 & \cdots & 0\\ 0 & \Gamma_{1}^{2} & \cdots & 0\\ \vdots & \vdots & \ddots & \vdots \\ 0 & 0 & \cdots & \Gamma_{1}^{U} \end{array}\right]A,P_{U}(\theta_{1}^{1},\theta_{1}^{2},\cdots ,\theta_{1}^{U})⊛A,\;\left[\begin{array}{cccc} \Gamma_{2}^{1} & 0 & \cdots & 0\\ 0 & \Gamma_{2}^{2} & \cdots & 0\\ \vdots & \vdots & \ddots & \vdots \\ 0 & 0 & \cdots & \Gamma_{2}^{U} \end{array}\right],P_{U}(\theta_{2}^{1},\theta_{2}^{2},\cdots ,\theta_{2}^{U}),\cdots ,\left[\begin{array}{cccc} \Gamma_{n}^{1} & 0 & \cdots & 0\\ 0 & \Gamma_{n}^{2} & \cdots & 0\\ \vdots & \vdots & \ddots & \vdots \\ 0 & 0 & \cdots & \Gamma_{n}^{U} \end{array}\right],P_{U}(\theta_{n}^{1},\theta_{n}^{2},\cdots ,\theta_{n}^{U})),$$

where $A\in R^{Ud_{0}\times d_{0}}$ satisfy

$$A=\left[\begin{array}{c} I_{d0}\\ \vdots \\ I_{d0} \end{array}\right].$$

Setting

$$z_{0}=\vartheta ,z_{1}=\left[\begin{array}{cccc} \Gamma_{1}^{1} & 0 & \cdots & 0\\ 0 & \Gamma_{1}^{2} & \cdots & 0\\ \vdots & \vdots & \ddots & \vdots \\ 0 & 0 & \cdots & \Gamma_{1}^{U} \end{array}\right]Az_{0}+[F_{\mu }(P_{U}(\theta_{1}^{1},\theta_{1}^{2},\cdots ,\theta_{1}^{U})⊛A)](z_{0}),=\left[\begin{array}{cccc} \Gamma_{1}^{1} & 0 & \cdots & 0\\ 0 & \Gamma_{1}^{2} & \cdots & 0\\ \vdots & \vdots & \ddots & \vdots \\ 0 & 0 & \cdots & \Gamma_{1}^{U} \end{array}\right](Az_{0})+F_{\mu }[(P_{U}(\theta_{1}^{1},\theta_{1}^{2},\cdots ,\theta_{1}^{U})](Az_{0}),=\left[\begin{array}{cccc} \Gamma_{1}^{1} & 0 & \cdots & 0\\ 0 & \Gamma_{1}^{2} & \cdots & 0\\ \vdots & \vdots & \ddots & \vdots \\ 0 & 0 & \cdots & \Gamma_{1}^{U} \end{array}\right]\left[\begin{array}{c} \vartheta \\ \vdots \\ \vartheta \end{array}\right]+F_{\mu }[(P_{U}(\theta_{1}^{1},\theta_{1}^{2},\cdots ,\theta_{1}^{U})]\left[\begin{array}{c} \vartheta \\ \vdots \\ \vartheta \end{array}\right].$$

It is relevant with the problem in Lemma 3.8, and

$$(R_{\mu }\Psi )(\vartheta )=((R_{\mu }\Theta^{1})(\vartheta ),(R_{\mu }\Theta^{2})(\vartheta ),\cdots ,(R_{\mu }\Theta^{U})(\vartheta )).$$

Now, let us consider

$$C(\theta_{i}^{1})=C(\theta_{i}^{2})=\cdots =C(\theta_{i}^{U})=(d_{i-1},\ell_{1}^{i},\ell_{2}^{i},\cdots ,d_{i})\in N^{L_{1}(\theta_{i}^{1})},\;\forall \;i\in \{1,2,\cdots ,n\}.$$

Thus,

$$P(\Psi )=\sum_{k=2}^{n}[P_{c}(P_{U}(\theta_{k}^{1},\theta_{k}^{2},\cdots ,\theta_{k}^{U}))+Ud_{k}Ud_{k-1}]+P_{c}(P_{U}(\theta_{1}^{1},\theta_{1}^{2},\cdots \theta_{1}^{U})⊛A)+d_{0}Ud_{1}=U\ell_{1}^{1}d_{0}+U\ell_{1}^{1}+[\sum_{j=2}^{L_{1}(\theta_{1}^{1})-1}(U\ell_{j}^{1}U\ell_{j-1}^{1}+U\ell_{j}^{1})]+Ud_{1}U\ell_{L_{1}(\theta_{1}^{1})-1}^{1}+Ud_{1}+d_{0}Ud_{1}\;\;+\sum_{k=2}^{n}[P_{c}(P_{U}(\theta_{k}^{1},\theta_{k}^{2},\cdots ,\theta_{k}^{U}))+Ud_{k}Ud_{k-1}]\leq U^{2}P_{c}(\theta_{1}^{1})+U^{2}d_{1}d_{0}+\sum_{k=2}^{n}[U^{2}P_{c}(\theta_{k}^{1})+U^{2}d_{k}d_{k-1}]\leq U^{2}[P_{c}(\theta_{1}^{1})+d_{1}d_{0}+\sum_{k=2}^{n}[P_{c}(\theta_{k}^{1})+d_{k}d_{k-1}]]=U^{2}E(\Theta^{1}).$$

Hence, the proof of Proposition 3.4 is complete. □

Theorem 3 Eq.(2.3)(a) and Eq.(2.3)(b) yielded the imprecise solutions $\vartheta_{N}(z)$ and $q_{N}(z)$, respectively. Then, ResNets can obtain $\vartheta_{N}(z)$ and $q_{N}(z)$, with polynomial complexity increasing with *X* (the total points taken in [0, T]) and N (the number of iterations of the estimate solution).

Proof Now, we demonstrate Theorem 3 by studying FNNs and ResNets. In particular, it means that we demonstrate the existence of a ResNets Ξ, with $\left(\vartheta_{0},q_{0}\right)$ being the input and $\left(\vartheta_{N},q_{N}\right)$ being the output, where $\vartheta_{n}={\left(\vartheta_{n}(z_{0}),\cdots ,\vartheta_{n}(z_{X+1})\right)}^{t},q_{n}={\left(q_{n}(z_{0}),\cdots ,q_{n}(z_{X+1})\right)}^{t}$, n=0,1,⋯,N. *X* points are calculated on the range [0, T], Δz =$\frac{T}{X+1}$, $z_{k}$ = kΔz,k=0,1,⋯,X+1. *X* is a huge number. The following is the proof structure:

We explain how ResNets *ϖ* transforms $\left(\vartheta_{n},q_{n}\right)$ into $\vartheta_{n+1}$, n=0,1,⋯,N−1, then show that the proof of above structure:

We prove how a ResNets *ϖ* transforms $\left(\vartheta_{n},q_{n}\right)$ to $\vartheta_{n+1}$. Then the input of *ϖ* is

$${\left(\vartheta_{n}(z_{0}),\cdots ,\vartheta_{n}(z_{X+1}),q_{n}(z_{0}),\cdots ,q_{n}(z_{X+1})\right)}^{t},$$

and the output is

$${\left(\vartheta_{n+1}(z_{0}),\cdots ,\vartheta_{n+1}(z_{X+1})\right)}^{t}.$$

The ResNets *ϖ* structure is depicted in Fig. 2. Referring to Fig. 2, we can gain a clear understanding of the steps involved in designing and implementing ResNets, allowing us to effectively visualize and comprehend the architecture's components and their interactions. ResNets *ϖ* contains a shortcut Γ and four FNNs including $\Theta_{1},\theta_{sub},\theta_{LRI}\;and\;\theta_{s}$. We then demonstrate how to design *ϖ* in the following steps.

**Figure 3:**
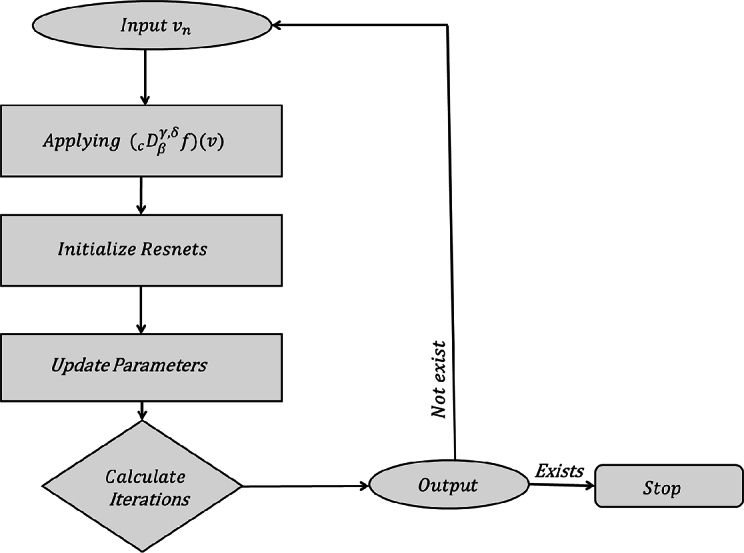
Flowchart illustrations of VI Method for ResNets.
Step 2 (Generating $\theta_{sub}$): Set FNNs $\theta_{sub}$= $\left(A_{sub},B_{sub}\right)$, where $A_{sub}=(I,-I)\in R^{\left(X+2)\times 2(X+2\right)}$ and $B_{sub}={\left\{0,\cdots ,0\right\}}^{t}\in R^{X+2}$. Now,
  $$(F_{\mu }\theta_{sub})(\vartheta ,y)=\vartheta -y,\;\forall \;\vartheta ,y\in R^{X+2},$$
  when input is
  (Dβγ,δcf(z0),⋯,Dβγ,δcf(zX+1),qn(z0),⋯,qn(zX+1))t,
  the output is
  (Dβγ,δcf(z0)−qn(z0),⋯,Dβγ,δcf(zX+1)−qn(zX+1))t.
Step 3 (Generating $\theta_{LRI}$): There exists a FNNs $\theta_{LRI}$ such that
  $$(F_{\mu }\theta_{LRI})(f)=I_{\beta }^{\gamma ,\delta }f,\;\;\forall f={\left(f(z_{0}),\cdots ,f(z_{X+1})\right)}^{t}.$$
  The left EKFI can be discretized as
  $$I_{\beta }^{\gamma ,\delta }f(\vartheta )=\frac{\beta \vartheta_{k}^{-\beta (\gamma +\delta )}}{\Gamma (\delta )}\int_{0}^{\vartheta_{k}}{\left(\vartheta_{k}^{\beta }-z^{\beta }\right)}^{\delta -1}z^{\beta (\gamma +1)-1}f(z)dz\approx \frac{\beta \vartheta_{k}^{-\beta (\gamma +\delta )}}{\Gamma (\delta )}\Delta \vartheta [{\left(\vartheta_{k}^{\beta }\right)}^{\delta -1}.0+{\left(\vartheta_{k}^{\beta }-\vartheta_{1}^{\beta }\right)}^{\delta -1}.\vartheta_{1}^{\beta (\gamma +1)-1}f(\vartheta_{1})+\cdots \;+{\left(\vartheta_{k}^{\beta }-\vartheta_{k-1}^{\beta }\right)}^{\delta -1}\vartheta_{k-1}^{\beta (\gamma +1)-1}f(\vartheta_{k-1})].$$
  Then there exists a FNNs $\theta_{LRI}$ = $\left(A_{LRI},B_{LRI}\right)$ with the input ${\left[f(\vartheta_{0}),f(\vartheta_{1}),\cdots ,f(\vartheta_{X+1})\right]}^{t}$ and output ${\left[I_{\beta }^{\gamma ,\delta }f(\vartheta_{0}),\cdots ,I_{\beta }^{\gamma ,\delta }f(\vartheta_{X+1})\right]}^{t}$,
  where,
  $$A_{LRI}=\frac{\beta \Delta \vartheta \vartheta_{k}^{-\beta (\gamma +\delta )}}{\Gamma (\delta )}\left[\begin{array}{ccccc} 0 & 0 & 0 & \cdots & 0\\ 0 & 0 & 0 & \cdots & 0\\ 0 & {\left(\vartheta_{2}^{\beta }-\vartheta_{1}^{\beta }\right)}^{\delta -1}\vartheta_{1}^{\beta (\gamma +1)-1} & 0 & \cdots & 0\\ \vdots & \vdots & \vdots & & \vdots \\ 0 & {\left(\vartheta_{X}^{\beta }-\vartheta_{1}^{\beta }\right)}^{\delta -1}\vartheta_{1}^{\beta (\gamma +1)-1} & 0 & \cdots & 0\\ 0 & {\left(T^{\beta }-\vartheta_{1}^{\beta }\right)}^{\delta -1}\vartheta_{1}^{\beta (\gamma +1)-1} & 0 & \cdots & 0 \end{array}\right]\in R^{\left(X+2)\times (X+2\right)}.$$
  Choose β(γ+1)>1, and $B_{LRI}={\left(0,0,\cdots ,0\right)}^{t}\in R^{X+2}$, such that
  $$\left[\begin{array}{c} I_{\beta }^{\gamma ,\delta }f(\vartheta_{0})\\ \vdots \\ I_{\beta }^{\gamma ,\delta }f(\vartheta_{X+1}) \end{array}\right]=A_{LRI}\left[\begin{array}{c} f(\vartheta_{0})\\ \vdots \\ f(\vartheta_{X+1}) \end{array}\right]+B_{LRI}.$$
  Thus when input of $\theta_{LRI}$ is
  (Dβγ,δcf(z0)−qn(z0),⋯,Dβγ,δcf(zX+1)−qn(zX+1))t,
  the output is
  (Iβγ,δ[Dβγ,δcf(z0)−qn(z0)],⋯,Iβγ,δ[Dβγ,δcf(zX+1)−qn(zX+1)])t.
Step 4 (Generating $\theta_{s}$): Consider FNNs $\theta_{s}$ = $\left(A_{s},B_{s}\right)$, where $A_{s}=-I\in R^{\left(X+2)\times (X+2\right)}$, and $B_{s}={\left\{0,\cdots ,0\right\}}^{t}\in R^{\left(X+2\right)}$, then
  $$(R_{\mu }\theta_{s})(\vartheta )=-\vartheta ,\;\forall \;\vartheta \in R^{\left(X+2\right)}.$$
  Thus, the input is
  (Iβγ,δ[Dβγ,δcf(z0)−qn(z0)],⋯,Iβγ,δ[Dβγ,δcf(zX+1)−qn(zX+1)])t,
  and output is
  (−Iβγ,δ[Dβγ,δcf(z0)−qn(z0)],⋯,−Iβγ,δ[Dβγ,δcf(zX+1)−qn(zX+1)])t.
Step 5 (Generating *ϖ*): ∃ a FNNs $\omega =\theta_{s}•\theta_{LRI}•\theta_{sub}•\Theta_{1}$ from Lemma 3.3, with the input
  $${\left(f(z_{0}),\cdots ,f(z_{X+1}),q_{n}(z_{0}),\cdots ,q_{n}(z_{X+1})\right)}^{t},$$
  and the output
  (−Iβγ,δ[Dβγ,δcf(z0)−qn(z0)],⋯,−Iβγ,δ[Dβγ,δcf(zX+1)−qn(zX+1)])t,
  where *ω* = $\left(A_{\omega },B_{\omega }\right)$, $A_{\omega }$ = $A_{s}A_{LRI}A_{sub}A_{1}\in R^{\left(X+2)\times 2(X+2\right)}$,
  $B_{\omega }=A_{s}[A_{LRI}(A_{sub}B_{1}+B_{sub})+B_{LRI}]+B_{s}\in R^{X+2}$,
  C(ω)=((2X+4),(X+2)).
  Then, the ResNets *ϖ* is represented by *ϖ* = (Γ,ω), and $\Gamma =(I,O)\in R^{\left(X+2)\times (2X+4\right)}$ with $I,O\in R^{\left(X+2)\times (X+2\right)}$ and O is null matrix.
  Since
  $$(R_{\mu }\varpi )(\vartheta )=\Gamma \vartheta +F_{\mu }\omega (\vartheta ),\forall \;\vartheta \in R^{\left(X+2\right)},$$
  and output of *ϖ* is
  {f(z0)−Iβγ,δ(Dβγ,δcf(z0)−qn(z0)),⋯,f(zX+1)−Iβγ,δ(Dβγ,δcf(zX+1)−qn(zX+1))}t,
  the input is
  $${\left(f(z_{0}),\cdots ,f(z_{X+1}),q_{n}(z_{0}),\cdots ,q_{n}(z_{X+1})\right)}^{t},$$
  and the level of complexity of *ϖ* is
  $$P(\varpi )=2(X^{2}+4X+4)+(X+2)+2(X^{2}+4X+4)$$
  =(X+2)(4X+9).
   □

Step 1 (Generating $\Theta_{1}$): $\Theta_{1}$ is generated with FNNs $\theta_{Caputo}$ and $\theta_{1}$ and we first describe how to construct $\theta_{Caputo}$. There exists a FNNs ([24]) $\theta_{Caputo}$ such that
  (FμθCaputo)(f(ϑn))=(Dβγ,δcf)(ϑ).
  If δ∈(0,1), the Caputo fractional derivative can be discretized by L1 technique:
  (Dβγ,δcf)(ϑ)=(Iβγ+δ,n−δ∏k=0k=n−1(1+γ+k+zβddz)f)(ϑ).
  Let k=0,n=1 and γ=−1,
  (Dβγ,δcf)(ϑ)=Iβγ+δ,1−δ(1+γ+zβddz)f(ϑ),=βΓ(1−δ)ϑ−β(γ+1)∫0ϑ(ϑβ−zβ)−δzβ(γ+δ+1)−1((1+γ+zβddz)f)(z)dz=1Γ(1−δ)∫0ϑ(ϑβ−zβ)−δzβδ−1zddzf(z)dz=1Γ(1−δ)∫0ϑ(ϑβ−zβ)−δzβδddzf(z)dz.
  Particularly, βδ→β−1, β>1,
  (Dβγ,δcf)(ϑ)=1Γ(1−δ)∫0ϑn(ϑβ−zβ)−δzβ−1ddzf(z)dz=1Γ(1−δ)[∫0ϑ1(ϑnβ−zβ)−δzβ−1ddzf(z)dz+⋯+∫ϑn−1ϑn(ϑnβ−zβ)−δzβ−1ddzf(z)dz]=1Γ(1−δ)∑k=0n−1∫ϑkϑk+1(ϑnβ−zβ)−δzβ−1ddzf(z)dz.
  Now we replace first order derivative by forward difference quotient, and assume that $\vartheta_{n}=nh,\vartheta_{k+1}=(k+1)h,\vartheta_{k}=kh$, as follows:
  (Dβγ,δcf)(ϑn)=1Γ(1−δ)∑k=0n−1∫ϑkϑk+1(ϑnβ−zβ)−δzβ−1[f(zk+1)−f(zk)h]dz=1hΓ(1−δ)∑k=0n−1[f(zk+1)−f(zk)]∫ϑkϑk+1(ϑnβ−zβ)−δzβ−1dz=−1βh(1−δ)Γ(1−δ)∑k=0n−1[f(zk+1)−f(zk)][(ϑnβ−ϑk+1β)1−δ−(ϑnβ−ϑkβ)1−δ].=−1βh(1−δ)Γ(1−δ)∑k=0n−1[f(zk+1)−f(zk)]{[(nh)β−((k+1)h)β]1−δ−[(nh)β−(kh)β]1−δ}=−(hβ)1−δβh(1−δ)Γ(1−δ)∑k=0n−1[f(zk+1)−f(zk)]{[(n)β−(k+1)β]1−δ−[(n)β−(k)β]1−δ},
  from the property of gamma function (1−x)Γ(1−x)=Γ(2−x), we obtain
  (Dβγ,δcf)(ϑn)=hβ(1−δ)−1βΓ(2−δ)∑k=0n−1[f(zk+1)−f(zk)]{−[(n)β−(k+1)β]1−δ+[(n)β−(k)β]1−δ};β>0Dβγ,δcf(zk+1)=hβ(1−δ)−1βΓ(2−δ)∑k=0n−1bk[f(zk+1)−f(zk)],
  where,
  $$b_{k}=[-{\left(n^{\beta }-{\left(k+1\right)}^{\beta }\right)}^{1-\delta }+{\left(n^{\beta }-k^{\beta }\right)}^{1-\delta }],A_{Caputo}=\frac{h^{\beta (1-\delta )-1}}{\beta \Gamma (2-\delta )}\left[\begin{array}{cccccc} 0 & 0 & 0 & \cdots & 0 & 0\\ 0 & b_{0} & 0 & \cdots & 0 & 0\\ \vdots & \vdots & \vdots & & \vdots & \vdots \\ 0 & b_{X-1} & b_{X-2} & \cdots & b_{0} & 0\\ 0 & b_{X} & b_{X-1} & \cdots & b_{1} & b_{0} \end{array}\right]\left[\begin{array}{cccccc} 0 & 0 & 0 & \cdots & 0 & 0\\ -1 & 1 & 0 & \cdots & 0 & 0\\ \vdots & \vdots & \vdots & & \vdots & \vdots \\ 0 & 0 & 0 & \cdots & 1 & 0\\ 0 & 0 & 0 & \cdots & -1 & 1 \end{array}\right]\in R^{\left(X+2)\times (X+2\right)},B_{Caputo}=(0,\cdots ,0)\in R^{X+2}.$$
  Therefore,
  [Dβγ,δcf(z0)⋮Dβγ,δcf(zX+1)]=ACaputo[f(z0)⋮f(zX+1)]+BCaputo.
  When the input of $\theta_{Caputo}$ is
  $${\left[f(z_{0}),\cdots ,f(z_{X+1})\right]}^{t},$$
  the output is
  [Dβγ,δcf(z0),⋯,Dβγ,δcf(zX+1)]t.
  Thus, a FNNs, $\theta_{1}=(A_{1},B_{1})$, where $A_{1}=I\in R^{\left(X+2)\times (X+2\right)}$, I is the IM, $B_{1}={\left(0,\cdots ,0\right)}^{T}\in R^{\left(X+2\right)}$ and $(F_{\mu }\theta_{1})(q_{n})=q_{n}$, which makes the input and output both ${\left(q_{n}(z_{0}),\cdots ,q_{n}(z_{X+1})\right)}^{T}$.
  As, $L_{1}(\theta_{Caputo})$ = $L_{1}(\theta_{1})=1$, there exists a FNNs $\Theta_{1}$ = $P_{2}(\theta_{Caputo},\theta_{1})$,
  $C(\Theta_{1})=\{2(X+2),2(X+2)\}$. The terms $L_{1}$ and C can refer to Definition 3.1,
  when $\Theta_{1}=(A_{1},B_{1})$, then
  $$A_{1}=\left[\begin{array}{cc} A_{Caputo} & 0\\ 0 & A_{1} \end{array}\right],B_{1}=\left[\begin{array}{c} B_{Caputo}\\ B_{1} \end{array}\right],$$
  the output of $\Theta_{1}$ is
  (Dβγ,δcf(z0),⋯,Dβγ,δcf(zX+1),(qn(z0),⋯,qn(zX+1)))t,
  with the input
  $${\left(f(z_{0}),\cdots ,f(z_{X+1}),q_{n}(z_{0}),\cdots ,q_{n}(z_{X+1})\right)}^{t}.$$
  The flowchart shown in Fig. 3 visualizes the step-by-step process of ResNets, making it easy to grasp the concepts and methods for solving fractional derivatives. This flowchart illustrates a step-by-step process that clarifies the iterations used in ResNets and provides a clear understanding of how to calculate derivatives using this method. Moreover, this flowchart can be applied to subsequent steps, offering a comprehensive guide for understanding ResNets and solving derivatives.

**Figure 2:**
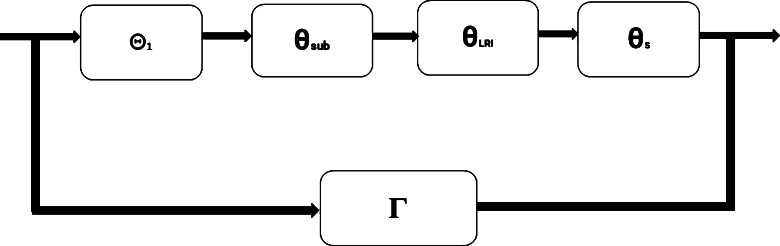
The ResNets structure *ϖ*. ResNets *ϖ* made up of a four FNNs and shortcut.

Now, some examples are given to understand the concepts.

## 4 Examples

Example 4.1 Consider the function f(x)=x for the problem:

(RμθCaputo)(f(xn))=(D1γ,0cf)(x).

Solution: To solve this example, we use Step 1 such that

Dβγ,δcf(tk+1)=hβ(1−δ)−1βΓ(2−δ)∑k=0n−1bk[f(tk+1)−f(tk)],

where

$$b_{k}=[-{\left(n^{\beta }-{\left(k+1\right)}^{\beta }\right)}^{1-\delta }+{\left(n^{\beta }-k^{\beta }\right)}^{1-\delta }],$$

by taking X=1,δ=0,γ=−1,β=1, and h=1, we find $b_{0}=1$, and $b_{1}=1$.

Therefore,

$$A_{Caputo}=\frac{1}{\Gamma (2)}\left[\begin{array}{ccc} 0 & 0 & 0\\ 0 & 1 & 0\\ 0 & 1 & 1 \end{array}\right]\left[\begin{array}{ccc} 0 & 0 & 0\\ -1 & 1 & 0\\ 0 & -1 & 1 \end{array}\right]=\left[\begin{array}{ccc} 0 & 0 & 0\\ -1 & 1 & 0\\ -1 & 0 & 1 \end{array}\right]$$

[Dβγ,δcf(t0)Dβγ,δcf(t1)Dβγ,δcf(t2)]=[000−110−101][t0t1t2]+[000]

$$=\left[\begin{array}{c} 0\\ -t_{0}+t_{1}\\ -t_{0}+t_{2} \end{array}\right].$$

Now, using Definition 2.1, Definition 2.3, we can solve the equation

Dβγ,δcf(t0)=0,f(t0)=t0=0.

Again for

Dβγ,δcf(t1)=t1−t0,(I1−1,2∏k=0k=n−1(1+γ+k+zβddz)f)(t)=t1−t0,I1−1,2[(zddz)(1+zddz)]f(t)=t1−t0.

On solving we get,

$$t_{1}-t_{0}=0,t_{1}=t_{0},$$

similarly for

Dβγ,δcf(t2)=t2−t0,

we get

$$t_{2}=t_{0}.$$

Now the example shows that when we take f(x)=x and apply the ResNets method to solve the fractional derivative, we obtain the solution $t_{0}=t_{1}=t_{2}=0$, which indicates a trivial or null solution, meaning that the solution is zero, signifying a breakdown in the ability to extract valuable information from the equation.

Example 4.2 This problem shows the result for 4×4 matrix when the function $f(x)=x^{2}$.

Solution: Using Step 1, by assuming δ=1/2,γ=0,β=1,h=1, and n=3, we have

D1γ,12cf(tk+1)=2π∑k=02bk[f(tk+1)−f(tk)],

where

$$b_{k}=[-{\left(n^{\beta }-{\left(k+1\right)}^{\beta }\right)}^{1-\delta }+{\left(n^{\beta }-k^{\beta }\right)}^{1-\delta }].$$

On putting values, we get

$$b_{k}=[{\left(3-k\right)}^{\frac{1}{2}}-{\left(3-(k+1)\right)}^{\frac{1}{2}}].$$

Therefore,

$$b_{0}=\sqrt{3}-\sqrt{2},b_{1}=\sqrt{2}-1,b_{2}=1.$$

Putting X=2, we obtain

ACaputo=hβ(1−δ)−1βΓ(2−δ)[00000b0000b1b000b2b1b0][0000−11000−11000−11]=1Γ(2−1/2)[000003−20002−13−20012−13−2][0000−11000−11000−11]=2π[00002−33−2001−222−1−33−20−12−222−1−33−2],[Dβγ,δct02Dβγ,δct12Dβγ,δct22Dβγ,δct32]=2π[00002−33−2001−222−1−33−20−12−222−1−33−2][t02t12t22t32]+[0000]=2π[0(2−3)t02+(3−2)t12(1−2)t02+(22−1−3)t12+(3−2)t22−t02+(2−2)t12+(22−1−3)t22+(3−2)t32].

Now,

Dβγ,δct02=0⇒t0=0.

Similarly for

Dβγ,δct12=2π((2−3)t02+(3−2)t12),

using Definition 2.1, Definition 2.3,

$$I_{1}^{1/2,5/2}((1+t\frac{d}{dt})(2+t\frac{d}{dt})(3+t\frac{d}{dt}))t_{1}^{6}=\frac{2}{\sqrt{\pi }}((\sqrt{2}-\sqrt{3})t_{0}^{2}+(\sqrt{3}-\sqrt{2})t_{1}^{2}),\frac{1}{\Gamma (5/2)x^{3}}\int_{0}^{x}{\left(x-t\right)}^{3/2}\sqrt{t}((1+t\frac{d}{dt})(2+t\frac{d}{dt})(3+t\frac{d}{dt}))t_{1}^{6}=\frac{2}{\sqrt{\pi }}((\sqrt{2}-\sqrt{3})t_{0}^{2}+(\sqrt{3}-\sqrt{2})t_{1}^{2}).$$

On solving we get,

(4.1) $$\frac{4}{3(\sqrt{\pi })x^{3}}\int_{0}^{x}{\left(x-t\right)}^{\frac{3}{2}}\sqrt{t}(6t_{1}^{6})dt=\frac{2}{\sqrt{\pi }}((\sqrt{2}-\sqrt{3})t_{0}^{2}+(\sqrt{3}-\sqrt{2})t_{1}^{2}).$$

Now, evaluate the definite integral $\int_{0}^{x}{\left(x-t\right)}^{\frac{3}{2}}\sqrt{t}dt$,

Let $t=x\sin^{2}u$; dt=(2xsin⁡ucos⁡u)du

$$=\int_{0}^{\frac{\pi }{2}}{\left(x-x\sin^{2}u\right)}^{\frac{3}{2}}{\left(x\sin^{2}u\right)}^{\frac{1}{2}}(2x\sin u\cos u)du.=2x^{3}\int_{0}^{\frac{\pi }{2}}(\cos^{4}u\sin^{2}u)du,=2x^{3}\int_{0}^{\frac{\pi }{2}}{\left(\frac{\cos2u+1}{2}\right)}^{2}(\frac{1-\cos2u}{2})du.$$

Further simplifying and evaluating the integral, we get,

(4.2) $$\frac{x^{3}}{16}{\left[\frac{\sin4u}{2}+2u+2\sin u-\frac{\sin6u}{6}+\frac{3\sin2u}{2}\right]}_{0}^{\frac{\pi }{2}}=\frac{\pi x^{3}}{16}.$$

We know $t_{0}=0$. So, from Eq.(4.1) and Eq.(4.2),

we get,

$$\frac{4}{3\sqrt{\pi }x^{3}}\times 6t_{1}^{6}\times \frac{\pi x^{3}}{16}=\frac{2}{\sqrt{\pi }}(\sqrt{3}-\sqrt{2})t_{1}^{2},t_{1}^{4}=\frac{4(\sqrt{3}-\sqrt{2})}{\pi },t_{1}=\sqrt[{4}]{\frac{4(\sqrt{3}-\sqrt{2})}{\pi }}.$$

Similarly for Dβγ,δct22,

$$\frac{\pi }{4}t_{2}^{6}=(2\sqrt{2}-1-\sqrt{3})t_{1}^{2}+(\sqrt{3}-\sqrt{2})t_{2}^{2}.$$

On putting the value of $t_{1}$, we get,

$$\frac{\pi }{4}t_{2}^{6}-(\sqrt{3}-\sqrt{2})t_{2}^{2}-[(2\sqrt{2}-1-\sqrt{3})\times \frac{2}{\sqrt{\pi }}\sqrt{\left(\sqrt{3}-\sqrt{2}\right)}]=0.$$

On solving,

$$t_{2}^{4}=\frac{0.63568}{4.7118},t_{2}=\sqrt[{4}]{\frac{0.63568}{4.7118}}.$$

And for Dβγ,δct32,

$$t_{3}=\sqrt[{4}]{\frac{0.63568}{4.7118}}.$$

Example 4.3 Consider the function $f(z)=\sin^{2}z+3\sin z+1$ for the problem:

(RμθCaputo)(f(zn))=(D2γ,0cf)(z).

Solution: To solve this example, we use Step 1 such that

Dβγ,δcf(tk+1)=hβ(1−δ)−1βΓ(2−δ)∑k=0n−1bk[f(tk+1)−f(tk)],

where

$$b_{k}=[-{\left(n^{\beta }-{\left(k+1\right)}^{\beta }\right)}^{1-\delta }+{\left(n^{\beta }-k^{\beta }\right)}^{1-\delta }],$$

by taking X=2,δ=0,γ=1,β=2, and h=1, we find

$$b_{0}=[-(3^{2}-1)+(3^{2})]=1,$$

and similarly,

$$b_{1}=3$$

$$b_{2}=5$$

Therefore,

$$A_{Caputo}=\frac{1}{2\Gamma (2)}\left[\begin{array}{cccc} 0 & 0 & 0 & 0\\ 0 & 1 & 0 & 0\\ 0 & 3 & 1 & 0\\ 0 & 5 & 3 & 1 \end{array}\right]\left[\begin{array}{cccc} 0 & 0 & 0 & 0\\ -1 & 1 & 0 & 0\\ 0 & -1 & 1 & 0\\ 0 & 0 & -1 & 1 \end{array}\right]=\frac{1}{2}\left[\begin{array}{cccc} 0 & 0 & 0 & 0\\ -1 & 1 & 0 & 0\\ -3 & 2 & 1 & 0\\ -5 & 2 & 2 & 1 \end{array}\right]$$

[Dβγ,δcf(z0)Dβγ,δcf(z1)Dβγ,δcf(z2)Dβγ,δcf(z3)]=12[0000−1100−3210−5221][2sin2⁡z0+3sin⁡z0+12sin2⁡z1+3sin⁡z1+12sin2⁡z2+3sin⁡z2+12sin2⁡z3+3sin⁡z3+1]+[0000]

(4.3) $$=\frac{1}{2}\left[\begin{array}{c} 0\\ -(2\sin^{2}z_{0}+3\sin z_{0}+1)+(2\sin^{2}z_{1}+3\sin z_{1}+1)\\ -3(2\sin^{2}z_{0}+3\sin z_{0}+1)+2(2\sin^{2}z_{1}+3\sin z_{1}+1)+(2\sin^{2}z_{2}+3\sin z_{2}+1)\\ -5(2\sin^{2}z_{0}+3\sin z_{0}+1)+2(2\sin^{2}z_{1}+3\sin z_{1}+1)+2(2\sin^{2}z_{2}+3\sin z_{2}+1)+(2\sin^{2}z_{3}+3\sin z_{3}+1) \end{array}\right].$$

Now, solving the first term Dβγ,δc(2sin2⁡z0+3sin⁡z0+1)=0 of the equation Eq.(4.3)

using Definition 2.1, Definition 2.3,

(4.4) $$I_{2}^{1,3}((2+t\frac{d}{dt})(3+t\frac{d}{dt})(4+t\frac{d}{dt}))(2\sin^{2}z_{0}+3\sin z_{0}+1)=0,I_{2}^{1,3}(24(2\sin^{2}z_{0}+3\sin z_{0}+1))=0\;2z_{0}^{-8}\int_{0}^{z_{0}}{\left(z_{0}^{2}-t^{2}\right)}^{2}t^{3}(24(2\sin^{2}z_{0}+3\sin z_{0}+1))dt=0\;\frac{2}{z_{0}^{8}}(24(2\sin^{2}z_{0}+3\sin z_{0}+1))\int_{0}^{z_{0}}{\left(z_{0}^{2}-t^{2}\right)}^{2}t^{3}dt=0.$$

Now, solving integration separately,

(4.5) $$I=\int_{0}^{z_{0}}{\left(z_{0}^{2}-t^{2}\right)}^{2}t^{3}dt,$$

we get,

$${\left[\frac{z_{0}^{4}t^{4}}{4}+\frac{t^{8}}{8}-\frac{2z_{0}t^{6}}{6}\right]}_{0}^{z_{0}}=\frac{1}{24}z_{0}^{8}.$$

Now, putting the value of Eq.(4.5) into Eq.(4.4) we have,

$$\frac{2}{z_{0}^{8}}(24(2\sin^{2}z_{0}+3\sin z_{0}+1))(\frac{1}{24}z_{0}^{8})=0$$

we can say that,

$$2\sin^{2}z_{0}+3\sin z_{0}+1=0.$$

So, the obtained value of $z_{0}$ will be,

$$z_{0}=-\frac{\pi }{2},-\frac{\pi }{6}.$$

Similarly, we can solve for all values of z using this approach. We've used a trigonometric example to demonstrate that neural networks can be applied to any type of equation to solve fractional derivatives. The trigonometric function serves as a clear illustration of the method, allowing for a precise understanding and accurate solution.

## 5 Conclusions

The approximation properties for residual networks in relation to the EKFD solution that have been achieved in this study by using the VI method. The VI method has primarily applied to FDEs containing EKFD and Caputo EKFD, and we solved the equations, including the left EKFD, using the VI technique to find the VI format for the solution of such equations and proved the convergence of the iteration method; hence, this iteration format is shown by using ResNets. The proof of Theorem 3 is concisely demonstrated in 5 steps, which involve utilizing ResNets to solve fractional differential equations. Fig. 1 provides a visual representation of the ResNets architecture, which serves to illuminate the specific steps employed in the proof. This research uses the VI method to show that using a neural network to approximate the EKFD solution is feasible. Finally, examples are provided to facilitate a deeper understanding of the method and to illustrate the step-by-step process for solving fractional derivatives, making the concepts more tangible and accessible.

## Additional information

No additional information is available for this paper.

## CRediT authorship contribution statement

**Sneha Agarwal:** Writing – original draft, Visualization, Validation, Software, Resources, Methodology, Investigation, Formal analysis, Conceptualization. **Lakshmi Narayan Mishra:** Writing – review & editing, Writing – original draft, Visualization, Validation, Supervision, Software, Resources, Methodology, Investigation, Formal analysis, Conceptualization.

## Declaration of Competing Interest

The authors declare that they have no known competing financial interests or personal relationships that could have appeared to influence the work reported in this paper.

## Data Availability

No data was used for the research described in the article.
